# The Role of Theatre-Based Methodologies as Complementary Educational Interventions in Continuing Nursing Education: A Scoping Review

**DOI:** 10.3390/ijerph22111657

**Published:** 2025-10-31

**Authors:** Giovanna Artioli, Andreina Saba, Laura Saladino, Allison Alberti, Laura Macchetti, Maria Chiara Bassi, Sara Falbo, Federica Dellafiore

**Affiliations:** 1 Department of Medicine and Surgery, University of Parma, 43125 Parma, Italy; 2 Department of Primary Health Care, Azienda USL-IRCCS of Reggio Emilia, 42122 Reggio Emilia, Italy; 3 Directorate of Nursing and Allied Health Professions, Azienda Socio Sanitaria Territoriale, Papa Giovanni XXIII, 24127 Bergamo, Italy; 4 Department of Molecular and Translational Medicine, University of Brescia, 25123 Brescia, Italy; 5 Maternal and Infantile Department, Azienda USL of Piacenza, 29121 Piacenza, Italy; 6 Medical Library, Azienda USL-IRCCS of Reggio Emilia, 42122 Reggio Emilia, Italy; 7 Directorate of Nursing and Allied Health Professions, Azienda Socio Sanitaria Territoriale of Melegnano and Martesana, 20077 Milan, Italy; 8 Department of Life Science, Health, and Health Professions, Link Campus University, 00165 Rome, Italy

**Keywords:** theatre-based education, continuing nursing education, complementary interventions, empathy, person-centred care, chronic illness, interprofessional learning

## Abstract

Theatre-based methodologies are increasingly recognized as complementary approaches that can enhance nurses’ empathy, communication, critical thinking, and person-centred care, all essential for managing chronic illnesses. This scoping review aimed to map and synthesize evidence on the application of theatre in continuing nursing education. A systematic literature search was conducted across five databases (PubMed, CINAHL, PsycINFO, Scopus, and Education Source) for publications in English and Italian up to 30 December 2024, supplemented by grey literature from ProQuest and reference screening via Google Scholar. Twenty-one studies met the inclusion criteria, identifying two main theatre methodologies, Forum Theatre and Drama, with four variations. These interactive methods were reported to foster reflective practice, enhance person-centred care, and improve interprofessional collaboration. Positive outcomes included improved nurse–patient relationships, quality of care, emotional engagement, cultural competence, teamwork, conflict management, and acceptance of diversity. Key facilitators were institutional support and active participation, while barriers included resistance to change and limited resources. These findings indicate that theatre-based education can serve as an effective complementary strategy to cultivate empathy, creativity, and reflective skills in continuing nursing education, supporting the development of holistic and patient-centred care practices. Further research is needed to explore the sustainability of acquired competencies in clinical practice.

## 1. Background

In recent years, there has been growing interest in adopting more comprehensive indicators of educational success, which go beyond academic outcomes to include students’ social, emotional, and relational development. Identifying educational practices that effectively achieve these ambitious goals remains challenging [1]. Within this context, arts-based pedagogy has emerged as an innovative, science-informed, complementary strategy, utilizing various art forms to facilitate learning across educational fields, with particular applications in healthcare and nursing education [2]. Among these arts, theatre stands out as a particularly effective methodology for promoting person-centred care, empathy, and professional development. Forum Theatre (or Forum Play) is an interactive theatrical approach in which participants actively engage in role-playing to explore real-life professional scenarios, reflect on decision-making, and experiment with alternative strategies. Drama, including Docudrama, involves scripted or semi-scripted performances that depict realistic events, allowing participants to critically analyze professional interactions and patient care situations. Artistic expression offers a non-invasive avenue to explore the intimate dimensions of individuals’ mental states, emotions, and lived experiences, fostering enhanced understanding of patients and supporting mindful, compassionate, and holistic care [3,4].

Theatre provides diverse learning opportunities for professional development [5,6,7]: theatre scenarios immerse participants in realistic situations to enhance experiential learning [8]; creative drama stimulates creativity and critical thinking [9,10]; interactive theatre promotes understanding of decision-making and conflict resolution [11,12,13]; improvisational theatre develops communication and teamwork skills [14,15]; and readers’ theatre engages participants in narrative reading to foster compassionate, holistic care approaches [3]. Recent evidence highlights theatre’s significant role in healthcare professional education, including nursing [2,14,16], enhancing communication, collaboration, reflective practice, self-awareness, and stress management [5,16,17].

These educational strategies can be considered complementary interventions, as defined in healthcare research, due to their measurable impact on professional competencies that directly influence patient care quality and outcomes. In healthcare research, “complementary interventions” refer to strategies that supplement traditional training approaches by targeting relational, reflective, and interpersonal competencies, enhancing professional skills that directly influence patient care. Evidence indicates that theatrical methods enhance empathy, promote active learning, stimulate critical reflection, and contribute to holistic, person-centred nursing practice [4]. Despite the time and resource required, these approaches are effective in cultivating skills essential for managing patients with chronic illnesses.

However, research on specific theatre-based methodologies remains fragmented, often focusing on university settings [18,19]. A notable gap exists regarding the application of theatre in continuing nursing education, which is critical for maintaining and enhancing clinical competencies among practicing nurses. As the demand for continuous professional development grows, there is an increasing need for practical, immediately applicable, and accessible learning strategies that cultivate soft skills, empathy, reflective thinking, and compassionate care [20].

Given the potential of theatre-based methodologies as complementary interventions to enhance nurses’ professional skills and, indirectly, patient outcomes in chronic care settings, further research is warranted. Previous reviews have primarily focused on arts- and theatre-based interventions in academic or undergraduate settings [18,19,21], often emphasizing theoretical frameworks and student learning outcomes. There is, however, a notable gap regarding the application of theatre methodologies in continuing nursing education for practicing professionals, particularly in relation to enhancing professional competencies, person-centred care, and the management of chronic conditions. This study aims to map and synthesize available evidence on the use of theatre in continuing nursing education, exploring pedagogical approaches, implemented methods, and contributions to professional development. Specifically, the review addresses the following questions:

(a) How are theatre methodologies structured and applied in continuing nursing education, and which approaches are most frequently used?
(b) Who are the key experts and participants involved in theatre-based education, and what insights do they provide on its impact?
(c) What are the perceived outcomes, barriers, and facilitators of implementing theatre methodologies in continuing nursing education, and how can these findings inform future development of complementary interventions in nursing practice?

## 2. Material and Methods

### 2.1. Study Design

A scoping review was conducted to map the existing literature, identify knowledge gaps, and explore potential opportunities for research and practical application of theatre as a complementary educational intervention in continuing nursing education. This methodology was guided by the Joanna Briggs Institute (JBI) guidelines [21,22], and is suitable for clarifying key concepts, definitions, and emerging practices in healthcare education [21,23]. The review was reported following the PRISMA extension for scoping reviews (PRISMA-ScR) checklist [24,25]. The study protocol was registered with the Open Science Framework (registration link: https://doi.org/10.17605/OSF.IO/AN9BW). This scoping review specifically addresses the potential of theatre-based methodologies to enhance nurses’ competencies relevant to the care of patients with chronic illnesses, emphasizing the role of complementary interventions in improving patient outcomes and quality of care.

### 2.2. Eligibility Criteria

Eligibility criteria were developed using the Participants, Concept, and Context (PCC) framework, following JBI guidance [21,22]. Studies were included if they reported on theatre-based educational interventions targeting practicing nurses or healthcare professionals engaged in continuing education, with potential implications for patient-centered care and chronic disease management. Chronic disease management was considered a contextual focus rather than a strict inclusion or exclusion criterion, reflecting its relevance for assessing professional competencies in ongoing nursing practice. The focus on chronic illness management was chosen because continuing nursing education interventions are frequently aimed at improving nurses’ competencies in managing patients with chronic conditions, where empathy, communication, and person-centred care are particularly crucial. Both qualitative and quantitative studies, as well as mixed-methods designs and relevant grey literature, were considered. Exclusion criteria comprised studies focusing solely on undergraduate education without clinical application, or those unrelated to theatre or complementary interventions. Book chapters were also excluded, as they are not consistently peer-reviewed, may lack standardized reporting, and thus do not ensure the same level of methodological rigor and reproducibility required for this review. Detailed inclusion and exclusion criteria are summarized in Table 1.

### 2.3. Search Strategy

The literature search was collaboratively designed within the research team, with guidance from an expert librarian (MCB). An initial exploratory search helped refine keywords, identify relevant synonyms, and determine potential limitations. The final search was conducted on 30 December 2024 across five electronic databases: PubMed, CINAHL, Scopus, PsycINFO, and Education Source (EBSCO). The search strategy combined keywords and MeSH terms including: “theatre” OR “drama” OR “interactive theatre” AND “nursing education” AND “continuing education” AND “complementary interventions” AND “chronic illness”.

The PubMed search strategy was: (((nurs* education[title/abstract]) AND (((psychodrama’[Mesh]) OR drama’[Mesh]) OR (theatrical[Title/Abstract] OR theatre[Title/Abstract] OR drama[Title/Abstract] OR theatre[Title/Abstract])) NOT (operating[title/abstract]). This strategy was subsequently adapted for each of the other databases with librarian support; the complete search strings are reported in Appendix A.

In addition, grey literature searches were performed via Google Scholar and ProQuest to capture professional reports, dissertations, and practice guidelines. For these platforms, free-text searches were conducted using combinations of the keywords “theatre”, “drama”, “nursing education”, and “continuing education”. As these sources do not allow for structured and fully reproducible search strings, the search was carried out in a transparent yet flexible manner, with relevant records screened manually. Citation searching was limited to the first ten included studies for feasibility reasons, in order to balance comprehensiveness with methodological rigor. This pragmatic decision ensured the retrieval of representative references while minimizing the risk of missing highly relevant publications.

### 2.4. Study Selection

Duplicate records were removed by the librarian, and results were imported into Rayyan, a web-based study screening management software [26]. Two reviewers (AS and SF) independently screened titles and abstracts for eligibility. The review process was conducted in a double-anonymous manner, meaning that both the authors and the reviewers remained blinded to each other’s identity, ensuring impartiality and minimizing potential bias in study selection and evaluation. Full texts of potentially relevant studies were then assessed by a team of reviewers (AS, SF, AA, LM, LS). Disagreements were resolved through discussion with senior researchers (GA and FD) until consensus was reached. The same process was applied to grey literature. The PRISMA flow chart details the selection process.

### 2.5. Data Extraction

Data were independently extracted by two reviewers (SF and AS) in a double-anonymized manner, using an Excel-based extraction tool developed for this review. The tool was piloted on the first five studies to ensure consistency. Extracted data included: authors, year, country, study design, aims, population, context, type of theatre intervention, trainers, trainees, and reported outcomes. Outcomes were categorized according to their relevance to nursing competencies, patient-centered care, and potential impacts on chronic disease management. This structured approach facilitates mapping of both pedagogical methods and their practical implications for complementary interventions in nursing. Table 2 presents the main characteristics of included studies.

Although formal quality appraisal is not mandatory in scoping reviews, the methodological characteristics of included studies were noted to contextualize findings and highlight potential limitations in the current evidence base.

### 2.6. Data Synthesis

Extracted data were synthesized using a narrative and thematic mapping approach, highlighting key concepts, trends, and gaps in theatre-based continuing education. This synthesis emphasizes connections between educational strategies, development of nurses’ complementary skills, and implications for patient care in chronic illness contexts, aligning with the focus of the Special Issue.

## 3. Results

**Figure 1:**
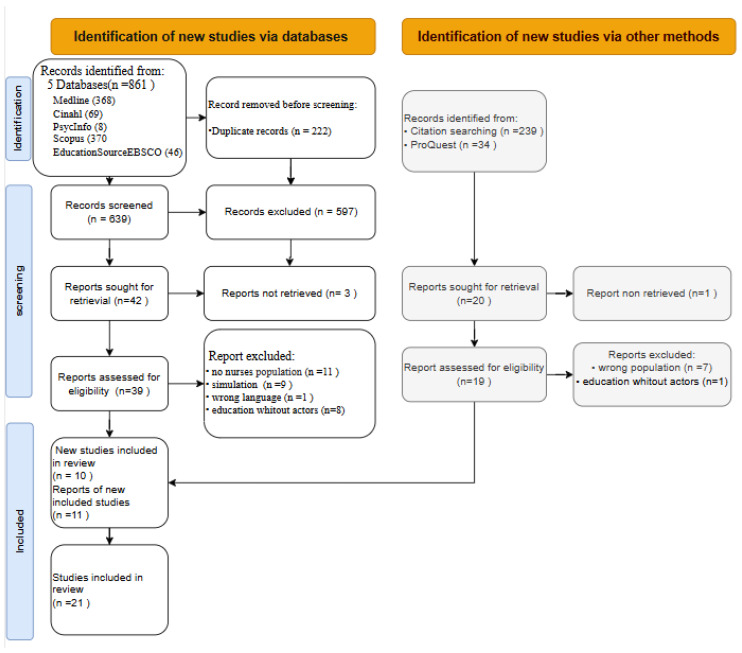
Flowchart of the study selection process. Preferred reporting items for systematic reviews and meta-analyses (PRISMA 2020 flow diagram) (Page et al., 2021) [4].

The initial bibliographic search across five databases yielded 861 records, which were reduced to 639 after removing duplicates. Forty-two sources were selected for full-text analysis, of which 3 were not retrievable, leaving 39 for eligibility assessment. Twenty-nine were excluded for not meeting inclusion criteria, resulting in 10 included articles. The grey literature search, using ProQuest and Google Scholar, identified 273 additional records. Screening by title and abstract led to 20 articles, of which one could not be retrieved; 19 were assessed in full, and 11 were included. Overall, 21 studies were included in this scoping review, following the PRISMA flowchart illustrated in Figure 1.

### 3.1. Features of Included Studies

The 21 included studies spanned the period 2010–2024, covering North America, Northern Europe, and Asia. Study designs were diverse: 13 qualitative, 1 mixed-methods, 6 quantitative, and 1 case study. Participants were mainly multi-professional healthcare providers, consistently including nurses; three studies focused exclusively on nurses [31,41,42]. Other professional roles involved across the included studies included physicians, physiotherapists, occupational therapists, psychologists, and social workers, reflecting the multi-professional nature of the interventions.

These studies explored theatre-based educational interventions aimed at improving professional skills, empathy, communication, and patient-centered care, with particular relevance to complementary strategies for managing chronic illness. The included studies employed a variety of evaluation methods, including questionnaires, interviews, reflective journals, and observational assessments, to assess outcomes such as empathy, communication skills, professional competence, and inter-professional collaboration. Most studies assessed outcomes immediately or shortly after the interventions, with only a few reporting longer-term follow-up, highlighting a gap in understanding the sustainability of the acquired skills.

The interventions addressed real-world healthcare challenges, such as dementia care, diabetes management, abuse recognition, and inter-professional communication, demonstrating the applicability of theatre beyond conventional academic settings. In terms of continuing professional development (CPD), the initiatives most commonly took the form of structured workshops, accredited training courses, or in-service training sessions organized by healthcare institutions, while some were integrated into broader interdisciplinary CPD programs. Rather than being a generic complementary intervention, theatre-based approaches in these studies were specifically implemented as experiential learning activities within CPD programs, aimed at enhancing professional skills, communication, empathy, and reflective practice among nurses and other healthcare professionals.

### 3.2. Theatre Methodologies

Two primary theatre-based methodologies emerged: **Forum Theatre/Forum Play** and **Drama**, which included Docudrama, Drama Workshops, the Change Cultural Studio Engagement Programme (CCSEP), and Ethnodrama (Table 3).

### 3.3. Forum Theatre or Forum Play

Forum Theatre, developed by Augusto Boal as part of the Theatre of the Oppressed, is a participatory approach where audience members, or “spect-actors,” actively intervene in staged conflicts to explore alternative solutions [40,41,45,46,47]. Forum Play, closely related, emphasizes interactive problem-solving in sensitive contexts, such as elder abuse, gender issues, and oppression [31,35,43]. These approaches were particularly effective in enhancing ethical reflection, moral reasoning, and inter-professional problem-solving, allowing participants to practice decision-making in a controlled yet realistic environment. Forum Theatre and Forum Play offer participants the opportunity to practice ethical decision-making, communication, and cultural sensitivity in a safe, guided setting, fostering skills directly applicable to clinical practice.

### 3.4. Drama-Based Interventions

Drama engages learners by transforming real-world experiences into performative narratives, using interviews, diaries, and observational data to create immersive scenarios. Drama-based methods, including Docudrama, Drama Workshops, CCSEP, and Ethnodrama, were especially effective in fostering empathic engagement, narrative understanding of patient experiences, and holistic care skills, complementing the reflective and decision-making benefits emphasized in Forum Theatre/Forum Play. This approach has been applied to complex healthcare contexts, such as dementia, diabetes, and brain injury rehabilitation [29,32,34,37,38]. Variations include:

Docudrama: dramatized real-life stories highlight key themes while preserving authenticity, engaging participants emotionally and cognitively with complex issues [42];

Drama Workshops: combine live performance and facilitated discussions, promoting patient empowerment, reflective thinking, and inter-professional collaboration [27];

CCSEP: integrates theatre and film to foster well-being, positive relationships, and person-centered care in care homes, using the PERMA framework [33];

Ethnodrama: transforms interviews and observations into live performances, allowing healthcare professionals to experience patients’ lived realities, fostering empathy and critical reflection in chronic disease care [28,36].

These approaches engage participants holistically, connecting emotional, cognitive, and relational dimensions of care, and demonstrate the value of complementary, person-centered interventions.

### 3.5. Trainers and Participants

The trainers’ profiles varied according to methodology. Forum Theatre/Forum Play sessions were typically led by theatre pedagogues and instructors, while drama-based interventions involved actors, theatre directors, and drama leaders, sometimes in collaboration with healthcare researchers. Participants included nurses, multi-professional teams, and in some cases, patients and families.

Collaboration between theatre and healthcare experts was critical to ensure interventions were contextually meaningful and clinically relevant [29,34,47]. For example:

**Forum Theatre**: participants actively engage in conflict resolution scenarios with guidance from theatre professionals [31,41].

**Drama**: participants explore ethical dilemmas and complex patient experiences, including brain injury rehabilitation and dementia care [32,37].

**CCSEP/Ethnodrama**: workshops integrate theatre and psychological principles to enhance relational care and empathy, involving healthcare teams, residents, and families [28,33].

### 3.6. Participants’ Outcomes

Across the included studies, theatre-based educational methods consistently demonstrated a positive impact on healthcare professionals, with a particularly strong effect on nurses’ development of core competencies relevant to complementary and person-centered care. Outcomes were not limited to knowledge acquisition but extended to behavioral change, ethical sensitivity, and relational skills that are essential in the management of chronic illness.

Forum Theatre/Forum Play proved especially effective in stimulating critical thinking and ethical reflection. By engaging participants in highly sensitive scenarios such as elder abuse, oppression, or gender-based violence, these methods fostered an environment where nurses could practice moral reasoning and test different communication strategies without real-world risks [31,35,40]. The iterative and participatory format enhanced participants’ sense of agency and responsibility, reinforcing their role as active problem-solvers in complex care situations.

Drama and Docudrama interventions contributed to heightened empathic engagement by immersing nurses and other professionals in dramatized patient experiences. These methods deepened understanding of the psychological and emotional dimensions of chronic illness, such as the lived realities of dementia or brain injury [29,37,42]. Outcomes included improved ability to interpret subtle patient cues, heightened sensitivity to non-verbal communication, and stronger capacity to deliver holistic, complementary care strategies.

Drama Workshops and CCSEP highlighted the importance of collective reflection and inter-professional dialogue. These approaches promoted cultural competence, empowerment, and inter-professional collaboration, particularly in long-term care and care home contexts [27,33]. Participants reported not only increased self-confidence but also a stronger capacity to engage in shared decision-making with patients and families, aligning with the principles of person-centered care.

Ethnodrama stood out for its ability to translate lived patient narratives into actionable professional insights. Nurses and multi-professional teams reported gaining a deeper appreciation of patients’ struggles, resilience, and coping strategies, which in turn reinforced their own commitment to relational and compassionate care [28,36]. This form of experiential learning had enduring effects on practice, as participants described being better equipped to engage in empathetic dialogue, anticipatory guidance, and reflective self-awareness in chronic illness management.

Taken together, these findings suggest that theatre-based interventions go beyond short-term learning outcomes: they cultivate reflective, empathetic, and ethically attuned practitioners who are better prepared to deliver sustainable, non-pharmacological care interventions in complex and chronic care settings.

### 3.7. Facilitators and Barriers

The successful implementation of theatre-based methods in healthcare education was influenced by several contextual facilitators and barriers.

Facilitators included:

- Institutional support and managerial engagement, which ensured that theatre interventions were recognized as legitimate educational strategies rather than extracurricular activities [28,41].
- Safe and inclusive learning environments, where participants felt encouraged to experiment, make mistakes, and reflect without fear of judgment [40]. This was especially important for sensitive themes like abuse, discrimination, or end-of-life care.
- Interdisciplinary collaboration, both in the design and facilitation of interventions, which enhanced the richness of the learning experience and promoted shared professional language across different healthcare roles [34,47].
- Interactive and reflective components, such as facilitated group discussions or post-performance debriefings, which helped participants translate theatrical experiences into concrete strategies applicable in clinical care. These reflective spaces were frequently cited as the moments when personal transformation and professional insight converged.

Barriers, on the other hand, highlighted the challenges of translating innovative methodologies into routine educational practice:

- Resource constraints (financial, logistical, and temporal) were among the most commonly reported barriers. Theatre-based interventions require specialized expertise, rehearsal time, and appropriate venues, all of which may exceed the budgets of conventional training programs [27,33].
- Resistance to innovation from staff and institutions occasionally limited uptake. Some participants and administrators perceived theatre as “soft” or non-academic, questioning its rigor compared with traditional didactic training. This skepticism could undermine engagement and institutional support [31].
- Time pressures in healthcare settings made it difficult to allocate sufficient time for immersive and reflective theatre-based sessions, particularly in acute or high-demand care contexts.

Despite these barriers, many studies emphasized that the transformative potential of theatre outweighed its challenges. Where leadership actively supported innovation and where reflective dialogue was built into the process, theatre-based interventions not only succeeded but also became catalysts for long-term cultural change in healthcare organizations.

## 4. Discussion

This scoping review offers a structured overview of theatre-based methodologies in continuing nursing education, addressing a gap in the existing literature. Although the use of the arts in education is widely recognized as a valuable strategy to develop essential soft skills, the specific role of theatre in ongoing nursing training has not yet been systematically explored. By synthesizing available evidence, this review provides a foundation for understanding how theatre-based approaches may support professional development and clinical practice, while acknowledging contextual limitations. Unlike previous works, which have primarily focused on academic or pre-licensure contexts, this review expands the perspective by highlighting the transformative potential of theatre in lifelong learning and professional development for nurses. The analysis considers not only educational benefits but also implementation challenges and facilitating factors, providing practical insights for healthcare organizations and educators.

The findings indicate that integrating theatre-based methodologies into nursing and interprofessional education can promote experiential learning, strengthen communication and empathy, and raise awareness of ethically complex issues. However, it is important to note that most studies assessed outcomes immediately or shortly after the interventions, and few reported longer-term follow-up, so the sustainability of these skills and the direct impact on patient care remain uncertain. These results are consistent with the literature that recognizes theatre as a vehicle for reflective and interactive learning environments [48,49]. Forum Theatre and Drama, in their various adaptations, have demonstrated the capacity to foster interactive reflection, preparing healthcare professionals to confront sensitive and emotionally demanding situations [32,37]. These approaches provide a psychologically safe environment where participants can explore emotions, uncertainties, and ethical dilemmas, supporting reflective practice rather than guaranteeing long-term behavioral change [29,42].

Forum Theatre and Forum Play were particularly effective in stimulating dialogue and fostering cultural awareness within healthcare organizations. Their participatory format allowed participants to reflect on issues such as racism, oppression, and gender-based violence [44,45,46,47]. The opportunity for participants to intervene directly during performances created occasions for experimenting with new communication strategies and problem-solving approaches in a protected setting, strengthening competencies with direct applicability in clinical practice [31,35,41,43]. The success of these interventions appears highly dependent on facilitation quality and participant engagement [48]. Similarly, Drama and Docudrama approaches facilitated empathic engagement by immersing participants in dramatized patient experiences, including dementia and brain injury rehabilitation [29,32,34,37,38,39]. These methods enhanced awareness of relational dynamics and person-centered care, although their long-term impact on professional practice remains to be evaluated [42].

Despite these benefits, barriers to implementing theatre-based methodologies persist, including resistance to innovation, resource constraints, and limited time for immersive sessions [28,33]. Sustainability also depends on institutional support and the creation of safe, inclusive learning environments. Facilitators included managerial commitment, integration into formal CPD programs, interdisciplinary collaboration, and facilitation techniques tailored to adult learners [28,29,34,40].

## 5. Limitations

Several limitations must be considered. First, the predominance of qualitative studies limits the generalizability of findings. Second, heterogeneity in intervention formats and facilitation methods makes it difficult to compare results or identify best practices. Most studies had short follow-up periods, so conclusions about lasting impact on professional practice and patient outcomes are tentative.

### 5.1. Future Research

Future studies should explore quantitative designs to measure the long-term impact of theatre-based education, and investigate how interventions can be effectively integrated into routine clinical practice. Research could also examine the role of organizational culture, levels of participant engagement, and the use of digital technologies or virtual simulations to enhance scalability and accessibility [34,44,47]. Multidisciplinary collaboration between theatre professionals, healthcare educators, and clinical teams is recommended to optimize educational and clinical outcomes.

### 5.2. Recommendations

Based on our synthesis of the literature, theatre-based methodologies should be thoughtfully integrated into continuing professional development programs for nurses, not as occasional or extracurricular activities, but as structured experiential learning opportunities that foster communication, empathy, and reflective practice. To maximize their impact, institutional support is crucial: leadership must provide adequate resources, endorse the educational value of theatre, and recognize its role in professional development. Facilitators should be trained both in theatre pedagogy and in healthcare education principles to ensure sessions are engaging, psychologically safe, and aligned with clinical realities. Moreover, future research should systematically evaluate the longer-term effects of theatre-based education, exploring not only professional competencies but also potential impacts on patient outcomes. Innovative approaches combining theatre with digital technologies and interdisciplinary collaboration may further enhance accessibility, scalability, and integration into routine healthcare education, ultimately contributing to a more reflective, empathetic, and competent nursing workforce.

## 6. Conclusions

This review reaffirms the value of theatre-based methodologies in nursing and interprofessional education, highlighting their potential to strengthen communication, empathy, and relationship management—core competencies for patient-centered care. While obstacles to adoption remain, the evidence suggests that institutional support and curricular integration can significantly facilitate broader and more systematic use of these innovative approaches. The transformative power of theatre, however, also raises a broader cultural and pedagogical question: are healthcare systems truly ready to embrace such a radical shift in training? Theatre challenges conventional forms of education by inviting participants to confront vulnerability, biases, and interpersonal dynamics. In this way, it disrupts traditional models centered exclusively on technical efficiency, fostering instead a more humanistic and person-centered orientation. Yet, to achieve this paradigm shift, it is necessary to overcome cultural, organizational, and economic barriers that currently limit diffusion. Ultimately, the challenge lies not only in recognizing the benefits of theatre-based education but in effectively embedding it into the landscape of continuing nursing education. Future studies will be essential to assess the long-term impact of these interventions and to identify strategies that optimize their integration across diverse healthcare settings.

## Figures and Tables

**Table 1:** Inclusion/exclusion criteria based on PCC.

Parameter	Inclusion Criteria	Exclusion Criteria
**Population**	Nurses (RN) attending continuing education courses, including in multi-professional groups	Others healthcare professionals, excluding nurses
**Concept**	Various theatrical methodologies using in continuing education	Simulations with equipment Educational events without actors, or with Simulations
**Context**	A wide range of nursing work settings and geographical areas.	
**Study design**	Primary research, both experimental and observational studies, qualitative study based on original data; Grey research; written in English, Italian, and Spanish-language; No restrictions were placed on publication year	Conferences abstract, systematic review, Expert opinions. Not research articles, Book chapters, written in other language by English, Italian, and Spanish.

**Table 2:** Main features of included studies.

Author/Year	Country	Study Design	Aim	Context	Trainers	Trainees	Outcomes
Abe et al., 2024 [27]	Japan	Mixed-methods design	To determine the impact of Diabetes Theater on participating healthcare professionals in terms of their knowledge and attitudes regarding patient empowerment.	The 57th Annual Scientific Meeting of the Japan Diabetes Society (2014)	Actors who perform a predefined theatrical performance	Nurses, dietitians, physicians, and pharmacists	Extending positive learning impact on participants’ perceptions of patient empowerment. Improving communication skills. Enhancing awareness of cultural diversity
Baillie et al., 2016 [28]	UK	Qualitative study, longitudinal design	To investigate staff perspectives of the effect of filmed drama on Dementia, on health professionals, their colleagues and the organization.	Two large Hospitals and Community Services	Theatre Players	Registered Nurses (RN) and other professions (clinical and not clinical)	Promoting a culture of reflection among healthcare professions to understand people with dementia. Improving empathy between healthcare professionals and people with dementia.
Bolmsjö et al., 2014 [29]	Sweden	Explorative Pilot study	To explore the use of drama as a tool to support reflection among staff working in the residential care of people with dementia.	A small Municipality in the South of Sweden	Theatre Director	10 Nurses	Improving reflection of Nurses about people with dementia
Brüggemann & Persson 2016 [30]	Sweden	Qualitative study: Constructivist Grounded Theory	To use Forum Play as a method to prevent and address abuse within these organizations.	A Nephrology Clinic	Professional drama instructors	12 Nurses and 2 other members of caring staff	Promoting reflection among caring staff about abuse on patients
Brüggemann et al., 2019 [31]	Sweden	Qualitative Action research	To analyze the conceptualizations, skills and tricks that care professionals use and develop to prevent and deal with situations they perceive as abusive to patients.	Hospice and Palliative Care Clinic (2016–2017)	Professional Theatre Instructors	Nurses, Physicians, administrator and managers	Understanding of how Health professionals make sense of and work against abuse in healthcare
Gjengedal et al., 2018 [32]	Norway	Qualitative study	To show how the theatre may yield new insight into living close to a person with dementia.	Local Dementia Associations and Nursing Home in three Norwegian Town	Regional Theatre in Norway	20 health professionals: Nurses, Nurses ‘aides, Music Therapist; 20 family members.	Transcending participants personal experiences and gaining new knowledge on dementia disease.
Guzmán et al., 2017 [33]	UK	Quasi-experimental case study	To evaluate the impact of the Culture Change Studio Engagement Program (CCSEP), a theatre and film-based staff training intervention, on staff working in Care Homes.	Two care Homes in UK	Professional Actors and expert in Positive Psychology	39 people from staff: Nurses, care and management staff	Improving a more positive interaction with the residents. Contributing to ameliorate the quality of life.
Hayes & Davis, 2014 [34]	USA	Descriptive study	To describe the use of the play format for education in hospital environments.	Medical Center	Playwright	Nurses	Developing empathy and reflective skills
Infanti et al., 2020 [35]	Sri Lanka	Qualitative study	To increase staff awareness of Abuse in Healthcare and promote taking action to reduce or prevent it.	Obstetrics and Gynecology Units at 3 Public Hospital in Colombo District	Theatre pedagogues	30 Nurses and 29 Physicians	Reducing the mistreatment of patients by healthcare providers.
Jonas-Simpson et al., 2012 [36]	Canada Ontario	Qualitative study	To explore the influence of an ethnodrama, called I’m Still Here (ISH), in changing understandings, images and actions about dementia and dementia care.	School of Continuing Education, Ryerson University in Toronto.	6 Actors	50 Healthcare professionals: Nurses, Physicians, Dietary and other professions	Improving relationship with patients Ameliorating humanization of care Changing about hope in healthcare professions
Kontos et al., 2010 [37]	Canada	Qualitative research	To introduce dementia practitioner’s person-centered care that emphasizes the notion of embodied selfhood (defined as non-verbal self-expression).	Two Nursing Home	Actors	Nurses and other professions	Contributing to promote patient centred approach into practice with people with dementia Gaining awareness that patients’ body movements express a language
Kontos et al., 2012 [38]	Canada	Qualitative study	To evaluate the short- and long-term impact of “After the Crash: A Play About Brain Injury,” a research-based drama designed to teach client-centered care principles to brain injury rehabilitation staff.	Neurorehabilitation Units in Ontario	Actors	33 healthcare professionals: 11 Nurses, 5 Occupational Therapy and other professions	Implementing a personalized care about Brain Injuri rehabilitation
Kontos et al., 2014 [39]	Canada	Qualitative study	To evaluate the impact of a Theatre intervention on emotion work practices in brain injury rehabilitation.	Brain Injury Rehabilitation Center	Professional acting troupe	Nurses, caring staff, patients and family	Improving therapeutic emotion work Ameliorating the culture of best practice
Ludvigsson et al., 2022 [40]	Sweden	Non randomised-stepped wedge trial	To determine the effectiveness of an educational intervention (Forum Theatre) on healthcare providers’ propensity to ask older patients questions about abusive experiences	Six hospitals in two regions (Region Östergötland and Region Jönköpings Län	Theatre pedagogues	Nurses, assistant nurses, physicians, occupational and physical therapists	Asking the olders patients questions about abuse
Meng & Sullivan 2011 [41]	Texas, USA	A survey	To support staff through interactive theatre in developing assertive communication skills to manage verbal abuse by family caregivers	Pediatric Unit	Professional Actor	Pediatric Nurses	Training assertiveness to for healthcare professionals Developing the emotional experience to cope with abuse by family members
Sorrell & Szweda 2015 [42]	USA	Descriptive study	To help ensure that new graduated nurses are prepared to provide safe and effective care for people with dementia	Cleveland Clinic	Theatre Director	60 New graduate Nurses	The Docudrama helps Nurses gin empathy about people with dementia Understanding the lived experience of family caregivers of people with dementia
Swahnberg et al., 2019 [43]	Sri Lanka	Descriptive study	To increasing staff awareness of obstetric violence and promote taking action to reduce or prevent it.	Obstetrics and Gynecology Units at 3 Public Hospital in Colombo District	Theatre pedagogues	Nurses, Physicians	Preventing and reducing abuse in healthcare
Tarasoff et al., 2014 [44]	Canada Ontario	Qualitative study	To determine how the assisted human reproduction (AHR) service providers meet the needs about lesbian, gay, bisexual, trans and queer (LGBTQ) patients.	Fertility Clinics	A person trained in Forum Theatre	30 healthcare providers: Nurses, obstetrics, Gynecologist and other professions	Changing in knowledge about this people Changing in comfort to work with LGBTQ by providers
Van Bewer et al., 2021/a [45]	Canada	Descriptive study	To describe the framework used in an anti-racist workshop (through sharing circles, image or forum theatre).	Faculty of Health Sciences of a Western Canadian University	Theatre Directors and Playwrights	7 participants: nurses and educators	Facilitating opportunities for intergroup dialogue. Increasing awareness of race and racism among White nurse educators
Van Bewer et al., 2021/b [46]	Canada	Qualitative Study with Thematic Analysis	To explore the priorities of Manitoba’s collaborative Indigenous education project with health care providers and students.	Faculty of Health Sciences of a Western Canadian University	Theatre Directors and Playwrights	6 Nurses and 2 other healthcare, with different racial origins	Learning the historical and contemporary context of Indigenous peoples’ lives.
Van Bewer et al., 2021/c [47]	Canada	Qualitative study with thematic analysis	To share participants’ experiences and reflections of TO (Theatre of the Oppressed) and FT (Forum Theatre) as pedagogies to illuminate the potential benefits and limitations in nursing education.	Faculty of Health Sciences of a Western Canadian University	Theatre Directors and Playwrights	6 nurses and 2 Other professions (white and indigenous)	Strengthening relationships and to practice vulnerability among health professionals

**Table 3:** Types of theatrical methods and definition.

Type of Methods	Definitions	Authors
**Forum Theatre or Play Forum**	**Forum Theatre**: is a form of participatory theatre involving actors and audience members, called ‘spect-actors’.	Van Bewer et al., 2021(a) [45] Van Bewer et al., 2021(b) [46] Van Bewer et al., 2021(c) [47] Tarasoff et al., 2014 [44] Ludvigsson et al., 2022 [40] Meng et al., 2011 [41]
	The **Forum Play** technique is very similar to forum theatre, requiring interaction and greater audience participation.	Brüggemann et al., 2019 [31] Swahnberg et al., 2019 [43] Bruggemann et al., 2016 [30] Infanti et al., 2020 [35]
**Drama**	Use qualitative research tools to construct dramas about complex experiences.	Gjengedal et al., 2018 [32] Hayes et al., 2014 [34] Bolmsjö et al., 2014 [29] Kontos et al., 2010 [37] Kontos et al., 2012 [38] Kontos et al., 2014 [39]
**a.** **DocuDrama**	A mix between drama and documentary	Sorrell et al., 2015 [42]
**b.** **Drama “ Theatres workshop”**	Interactive workshop	Abe et al., 2024 [27]
**c.** **Change cultural Studio Engagement programme (CCSEP)**	Theatre and film activity are using to improve communication	Guzmán et al., 2017 [33]
**d.** **Ethnodrama**	It combines elements of qualitative ethnographic research with artistic practices.	Baillie et al., 2016 [28] Jonas-Simpson et al., 2012 [36]

## Data Availability

No new data were created or analyzed in this study. Data sharing is not applicable to this article.

## Appendix A. Search Strategies

**DATABASES**	**Research Strategy**
Medline/PubMed	(((nurs* education[title/abstract]) AND (((psychodrama’[Mesh]) OR drama’[Mesh]) OR (theatrical[Title/Abstract] OR theatre[Title/Abstract] OR drama[Title/Abstract] OR theatre[Title/Abstract])) NOT (operating[title/abstract])
Cinahl	(TI nurs* education OR AB nurs* education) AND ((MH “Psychodrama”) OR (MH “Drama”) OR TI ( T h e a t r i c a l OR theatre OR drama OR theatre ) OR AB ( Theatrical OR theatre OR drama OR theatre )) NOT operating
PsycInfo	“nurs* education”.ab,ot. AND (exp Drama/ OR exp Psychodrama/ OR (Theatrical or theatre or drama).ab,ot.)
SCOPUS	( TITLE-ABS-KEY ( nurs* AND education ) AND TITLE-ABS-KEY (( theatrical OR theatre OR drama ) ) AND NOT TITLE-ABS-KEY ( operating ) )
Education source (EBSCO)	(TI nurs* education OR AB nurs* education) AND ( AB ( Theatrical OR theatre OR drama OR theatre ) OR TI ( Theatrical OR theatre OR drama OR theatre )) NOT operating
